# Supplementary material for: Seasonality in the migration and establishment of H3N2 Influenza lineages with epidemic growth and decline
Source: BMC Evol Biol. 2014 Dec 24;14:3. doi: 10.1186/s12862-014-0272-2 (PMC4316805; doi:10.1186/s12862-014-0272-2)
Supplement: Additional file 1 — Supplementary figures. [file 12862_2014_272_MOESM1_ESM.pdf]

SUPPLEMENTARY FIGURES

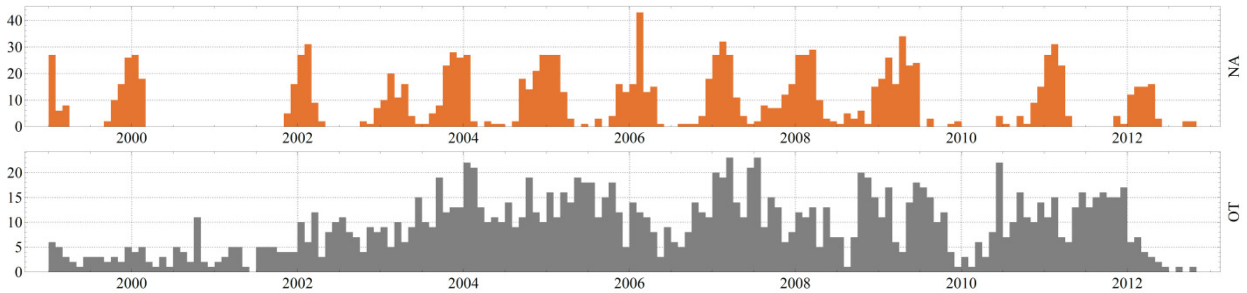


**Figure S1. Sequence Samples in NA and OT.** The monthly distribution of H3 and N2 sequence samples used in a partition of available sequences into North-America (NA), and a representative of the global community (OT).

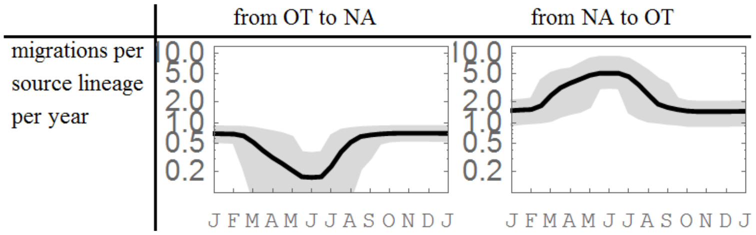


**Figure S2. Per-Lineage Migration Rates between NA and OT.** The median (black) and 90% Bayesian credible intervals (gray outline) of the migration model parameters (per-lineage migration rates) between North-America (NA), and a representative of the global community (OT).

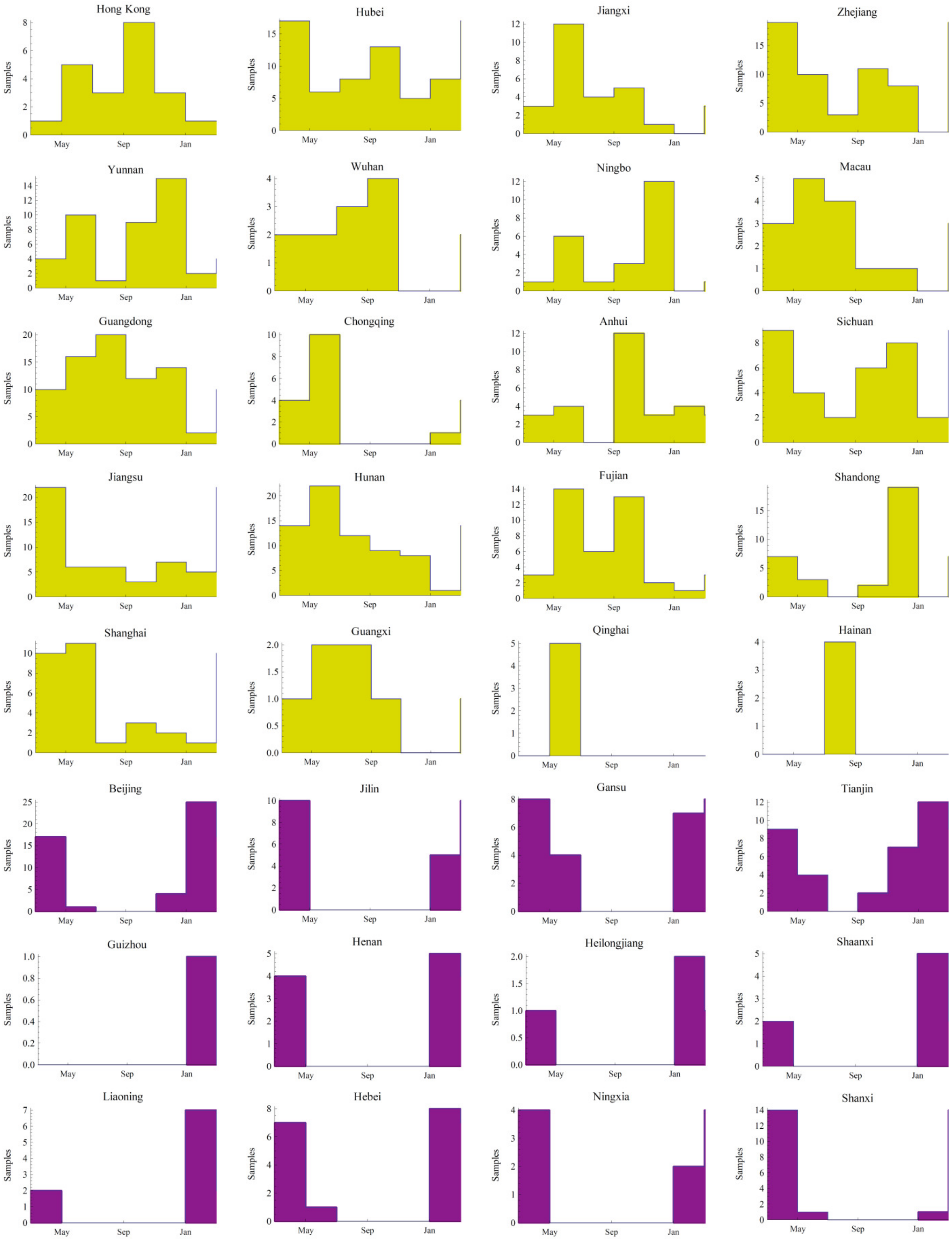


**Figure S3. Sequence Samples in Chinese Provinces and Several Large Cities.** The number of sequences collected in bimonthly partitions of the year was used to establish broad seasonal patterns in Chinese provinces and several large cities. (yellow, upper rows) Provinces classified as having a southern Chinese seasonality. (purple, lower rows) Provinces classified as having a northern Chinese H3N2 seasonality.

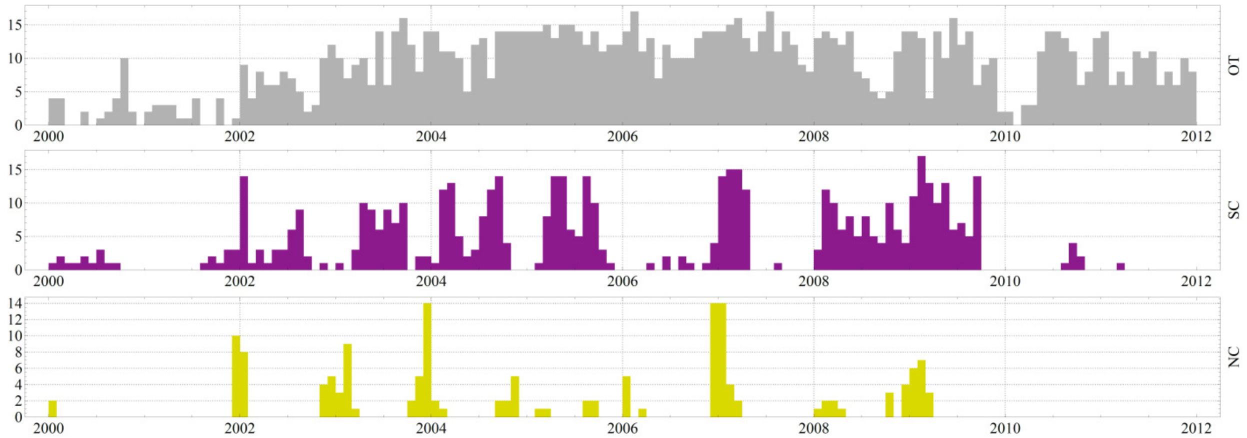


**Figure S4. Sequence Samples in SC, NC and OT.** The monthly distribution of H3 and N2 sequence samples used in a partition of available sequences into South-China (SC), North-China (NC) and a representative of the global community (OT).

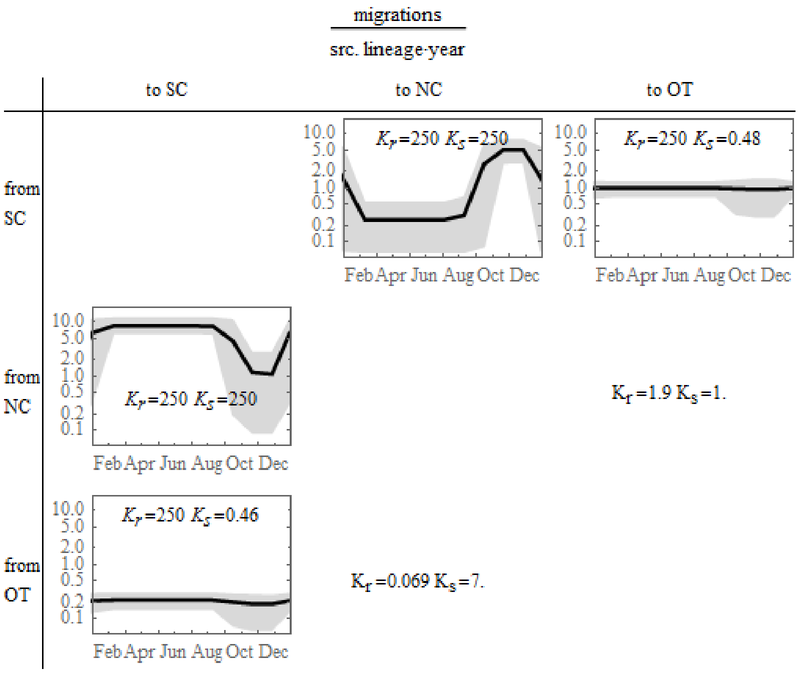


**Figure S5. Per-Lineage Migration Rates between SC, NC and OT.** The median (black) and 90% Bayesian credible intervals (gray outline) of migration model parameters (per-lineage migration rates) between North-China (NC), South-China (SC) and a representative of the global community (OT).

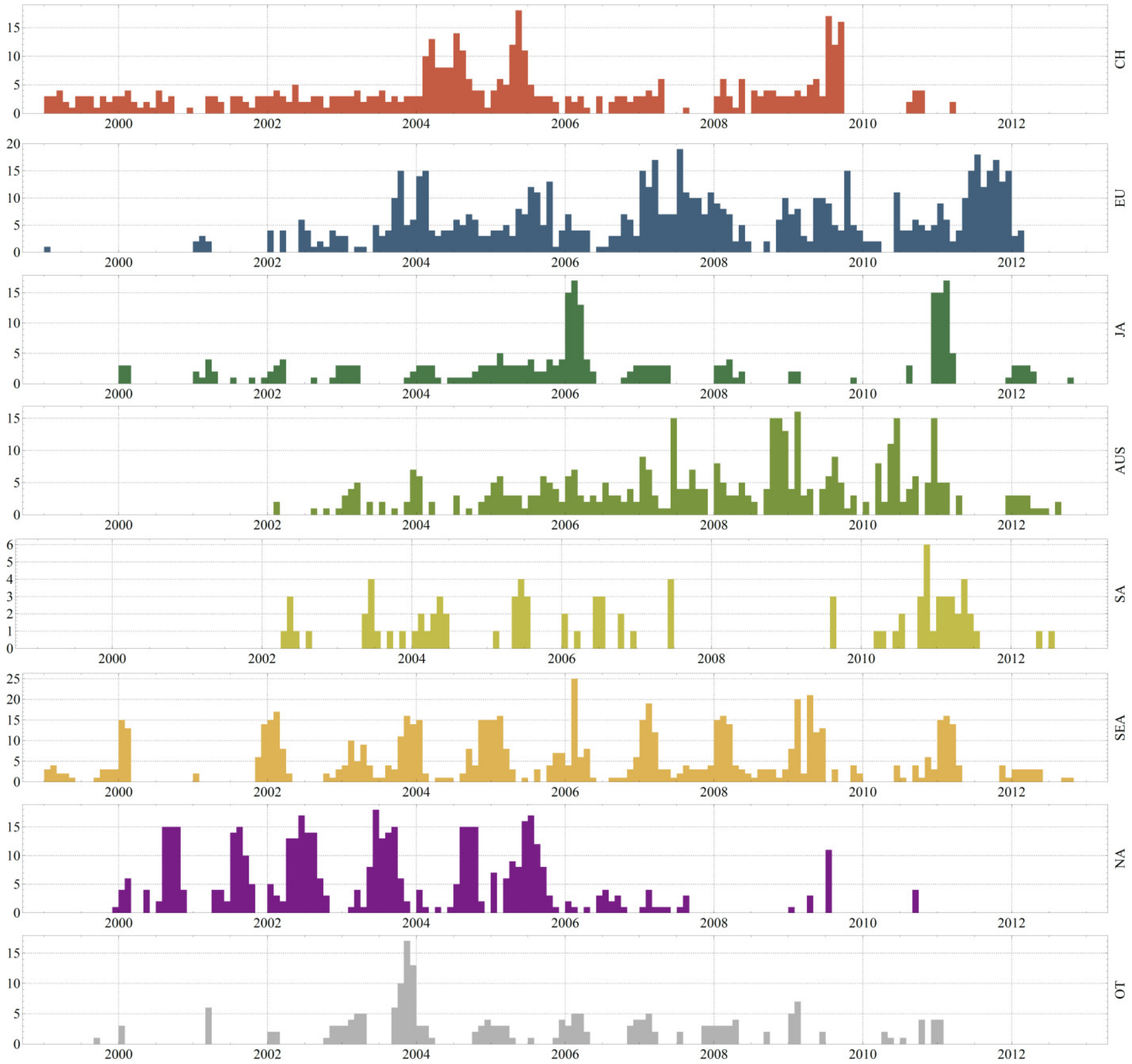


**Figure S6. Sequence Samples into Seven Global Communities.** The monthly distribution of H3 and N2 sequence samples used in a partition of available sequences into (CH – China, EU – Europe, JA – Japan, AUS – Oceania, SA - South-America, SEA - South East Asia, NA – North-America) and a representative collection of sequences sampled from other parts of the world designated as other (OT).

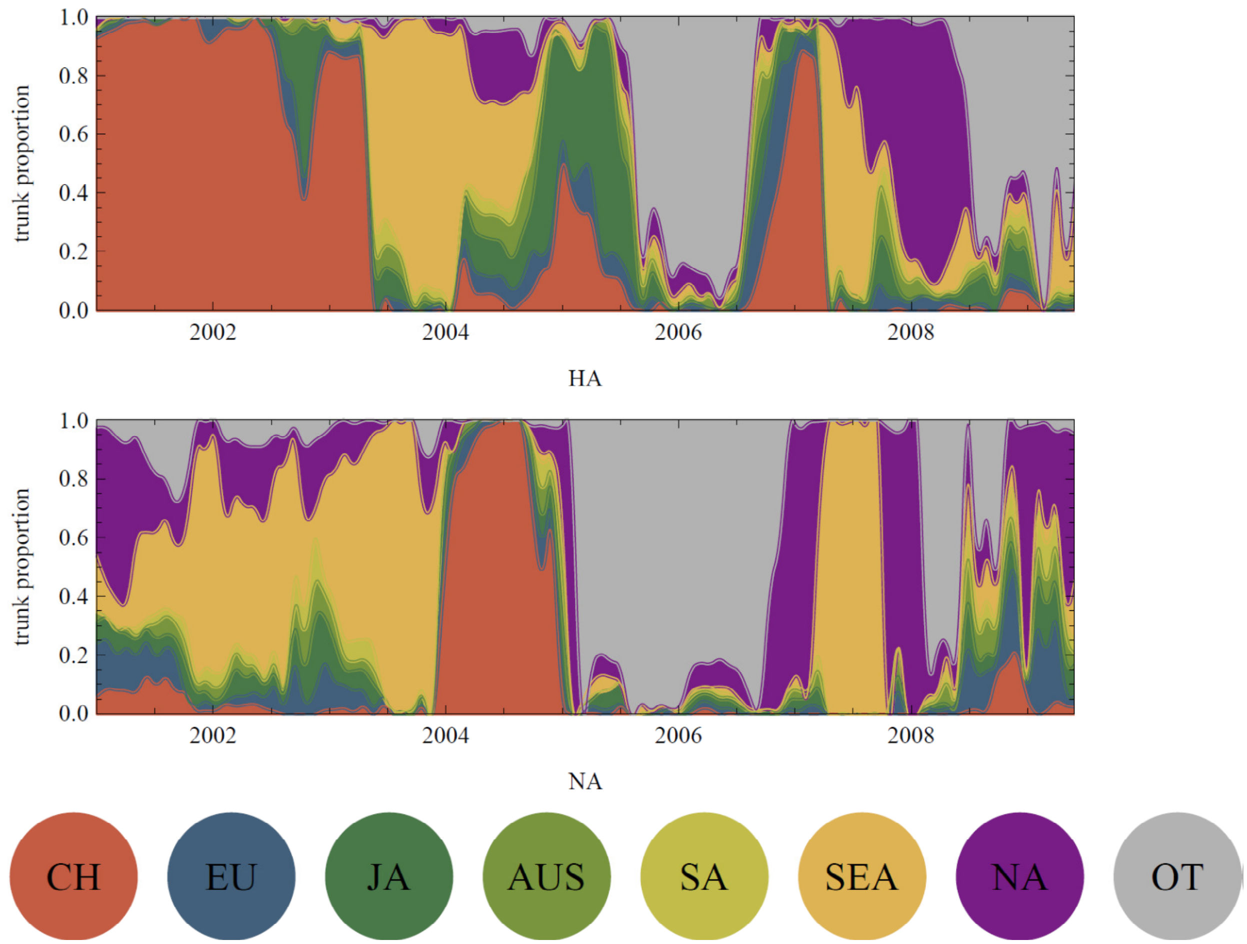


**Figure S7. The Inferred Location of the Global Trunk of the Hemagglutinin and Neuraminidase Proteins.** The percent of the global hemagglutinin (top) and neuraminidase (bottom) trunks inferred to be in the different global communities through time (CH – China, EU – Europe, JA – Japan, AUS – Oceania, SA - South-America, SEA - South East Asia, NA – North-America). Publically available human H3N2 hemagglutinin and neuraminidase sequence samples included in the analysis. A representative collection of sequences sampled from other parts of the world is designated as other (OT). For each protein the median proportion of the phylogenetic tree trunk in each deme through time is taken from the distribution of stochastically mapped trunk states given the sampled empirical posterior distribution of seasonal migration model parameters.

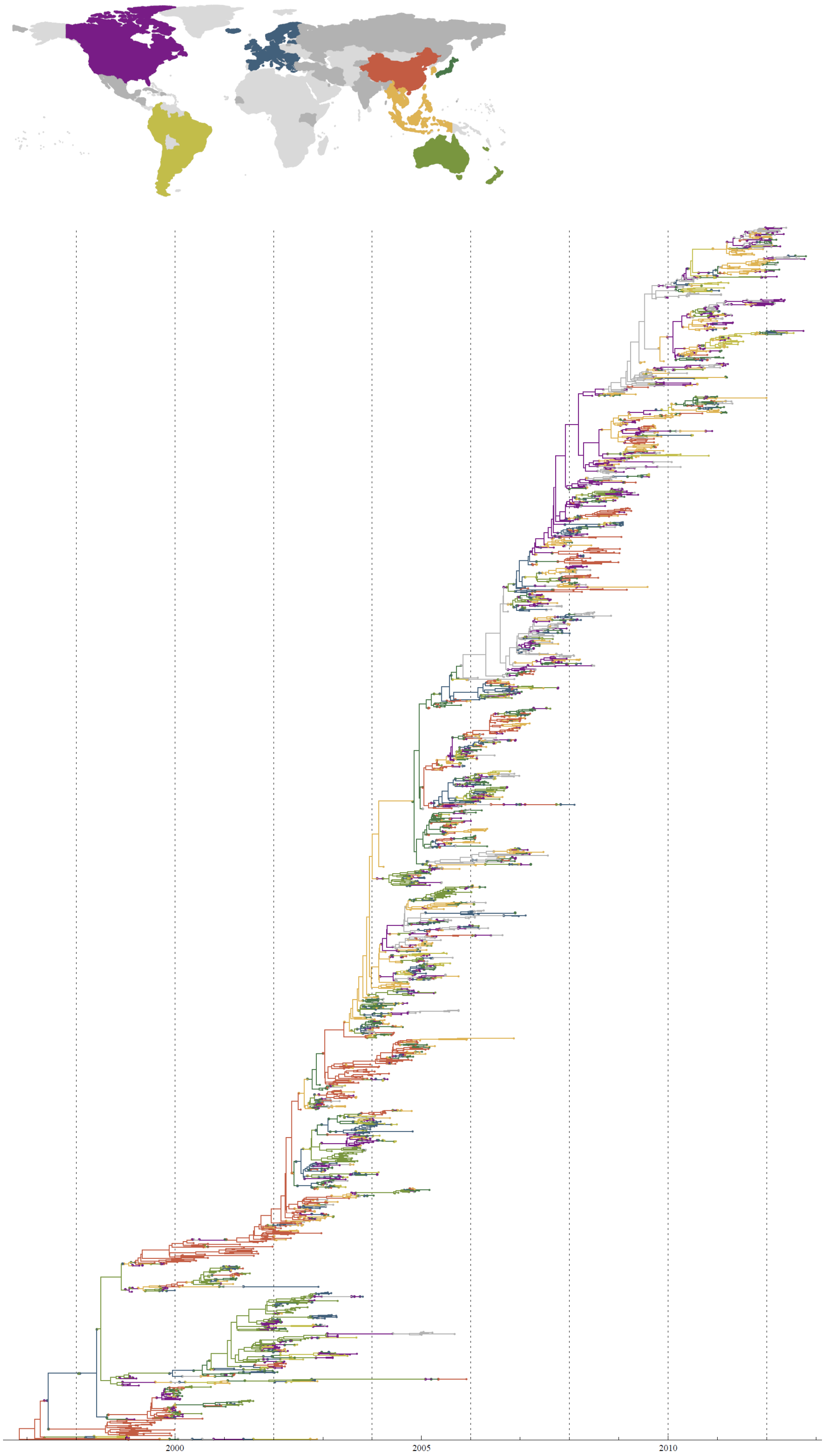


**Figure S8. A Single Stochastic Mapping of the Hemagglutinin Protein Tree.** A single stochastic mapping of the hemagglutinin tree branches. Branch parts inferred to be present in different geographical locations are colored according their community classification (map above). Migration events are visible in color transitions along branches.

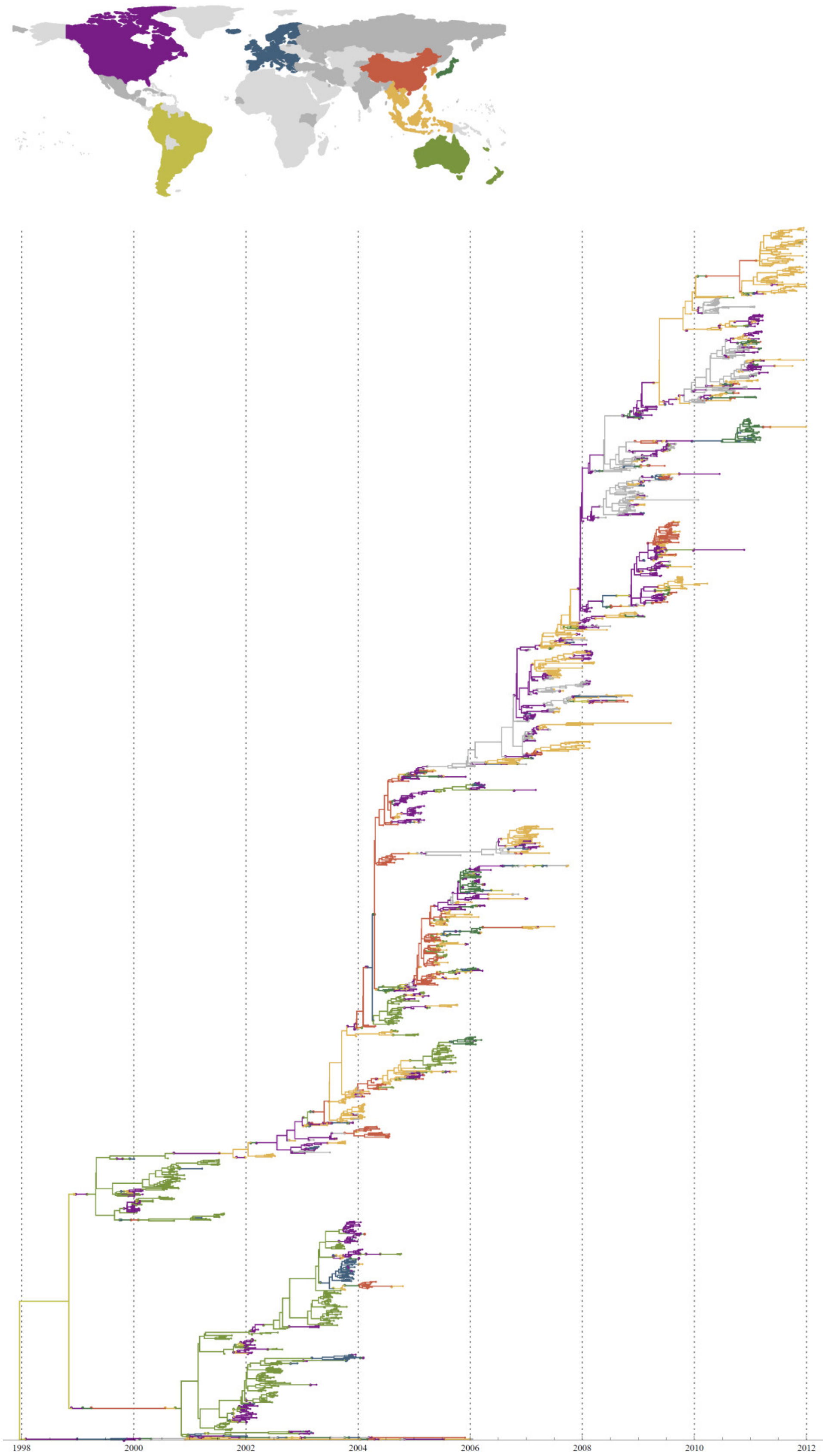


**Figure S9. A Single Stochastic Mapping of the Neuraminidase Protein Tree.** A single stochastic mapping of the neuraminidase tree branches. Branch parts inferred to be present in different geographical locations are colored according their community classification (map above). Migration events are visible in color transitions along branches.

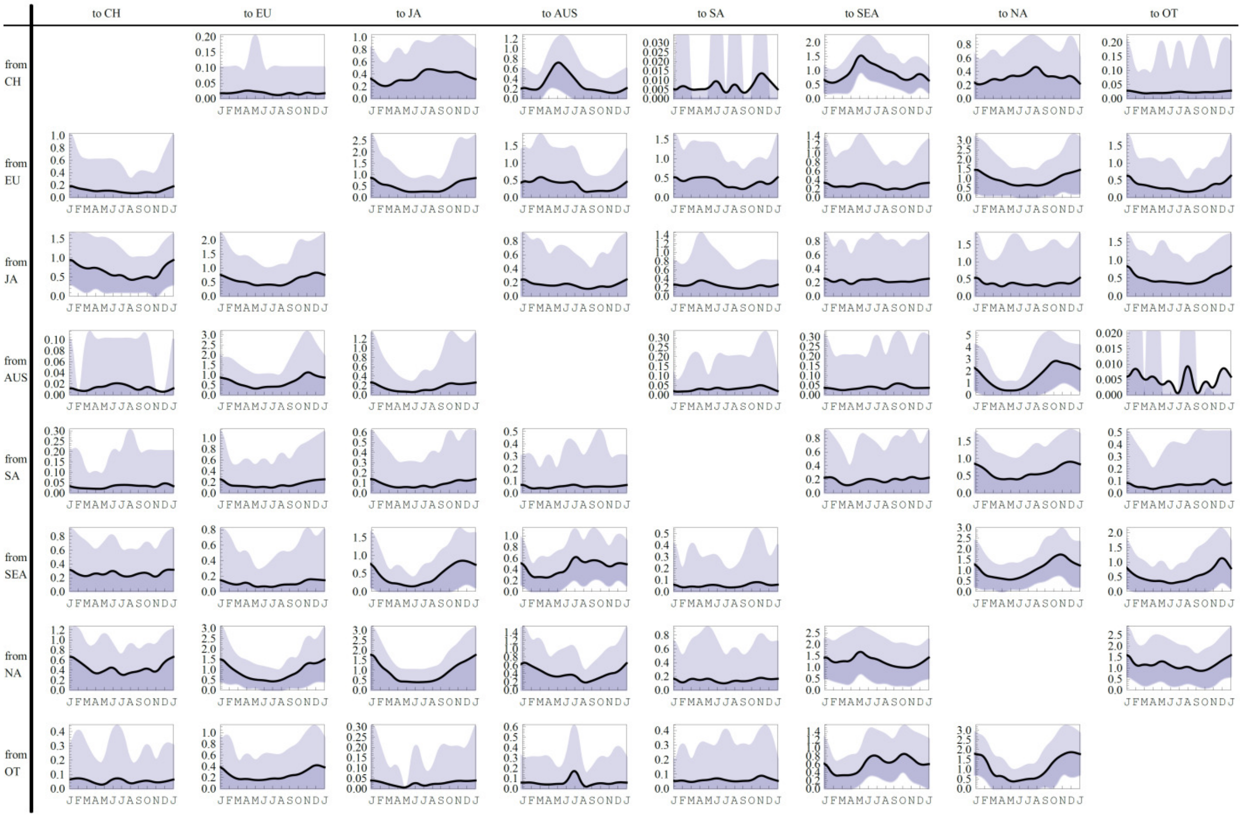


**Figure S10. Seasonal Migration between Seven Global Communities.** The inferred mean and 90% Bayesian credible interval (90% CI) of the number of migration events between 7 global communities through time (CH – China, EU – Europe, JA – Japan, AUS – Oceania, SA – South-America, SEA – South East Asia, NA – North-America). Inferred migrations to other (OT) regions not included in our classification appear on the rightmost column and bottom row.

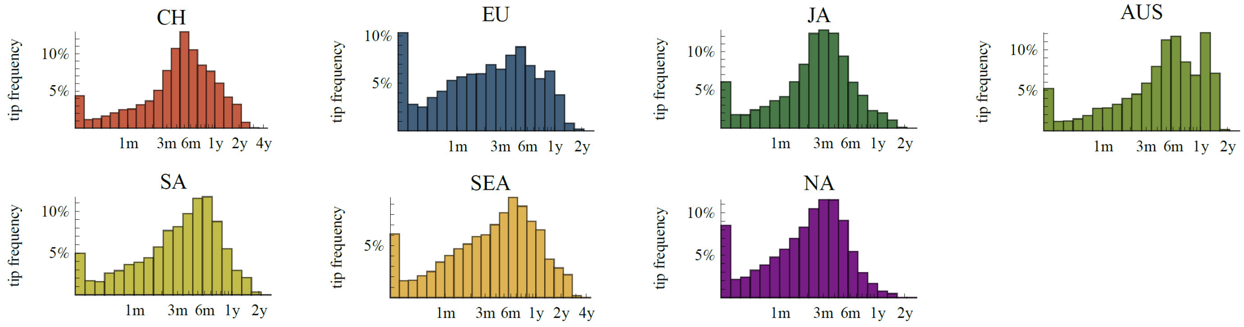


**Figure S11. Local Persistence in 7 Global Communities.** The inferred distribution of time for which samples sequences trace back their ancestry as remaining in the respective collection location (CH – China, EU – Europe, JA – Japan, AUS – Oceania, SA – South-America, SEA – South East Asia, NA – North-America).
