# Supplementary material for: Seasonality in the migration and establishment of H3N2 Influenza lineages with epidemic growth and decline
Source: BMC Evol Biol. 2014 Dec 24;14:3. doi: 10.1186/s12862-014-0272-2 (PMC4316805; doi:10.1186/s12862-014-0272-2)
Supplement: Additional file 2 — Supplementary Methods for Bayesian Inference and Stochastic Mapping of Seasonal Migration Processes from Phylogenetic Tree Distributions (SeasMig). [file 12862_2014_272_MOESM2_ESM.pdf]

Text S1: Supplementary Methods for Bayesian Inference and Stochastic Mapping of Seasonal Migration Processes from Phylogenetic Tree Distributions *(SeasMig)*

Supplement for: Seasonality in the migration and establishment of H3N2 Influenza with epidemic growth and decline (Zinder et al.)

In this supplement we describe in further detail the general Bayesian modeling approach, the mathematical details of the model, and the computational techniques used to perform inference and model selection. We demonstrate the method for several simulated scenarios in the context of seasonal migration.

Note: Sections 2.10-2.13 of this text partially overlap with [1] *supplementary-text and* [2]*, with author's (co-author Ed Baskerville) permission.*

1 Inference of migration processes from tree distributions

A variety of tools has been developed to generate phylogenetic trees from sequence data. Some are Bayesian in nature and provide a distribution of possible trees [3–5]. These trees are sampled according to their likelihood and according to the given prior probability for the parameters used when estimating this likelihood. A sample from this distribution is referred to as a sample from the empirical posterior distribution of trees. Other phylogenetic tree reconstruction tools capable of handling larger datasets e.g. [6, 7] often provide a single tree output or distributions which are based on randomizations of the data. It is very common for tree reconstruction to be the most computationally expensive part of phylogenetic analysis. This is the case since the number of possible trees grows super-exponentially in relation to the number of tips (or taxa).

In some cases it is possible to separate the inference of tree topology based on nucleotide data, from additional steps relating to the inference of phylogeography and phenotypic traits. This is the case when the contribution of these traits to the combined sequence and trait based tree likelihood is sufficiently small. In this case, the tree distribution is mostly defined based on sequence data and it can be further refined and used for trait based analysis [5]. If all the trees based on nucleotide data were enumerated, a step which is computationally infeasible for more than a few taxa, than an additional step involving inference based on traits will be mathematically equivalent to joint inference based on nucleotide and trait data, as long as the evolutionary models for traits and for individual nucleotide substitutions are independent. It is not easy to know exactly how many tree samples based on nucleotide data are required for trait based inference, but it is necessary that the sample should be large enough to capture the major different tree topologies.

In addition, given a distribution of trees, it is often useful to generate stochastic realizations of possible mutation and migration events as they occurred along the branches of the tree [8–10]. This can be done in a Bayesian manner, where for each tree topology coupled with a corresponding mutational and trait evolutionary model sampled form the empirical posterior distribution, a stochastic realization is generated.
Finally, the different genes, or proteins of an organism, may have alternative evolutionary histories. If the underlying migration processes is assumed to be the same, we can use information from multiple proteins together when inferring migration processes.

2 Implementation

2.1 *SeasMig*

We implemented in Java a tool (http://bitbucket.org/pascualgroup/seasmig) for migration model inference. This tool can also perform stochastic mapping, based on an initial distribution of trees and geographic annotations. Alternative migration model parameters can be inferred and compared by their marginal likelihoods including seasonal and epochal phylogeographic migration models. An empirical distribution of trees in nexus format is given as input. Our tool uses an MCMC to sample from a posterior distribution of model parameters and stochastically mapped migration events along branches and trunk lineages.

2.2 Bayesian Inference

In a Bayesian framework, both the data and the model parameters are assumed to be stochastic. Rather than finding the set of parameters that maximizes the likelihood of a particular observation, we estimate the distribution of the model parameters that can lead to the observed data. The probability of observing a specific set of model parameters θ conditioned on observing the data D is known as the *posterior* probability and can be written according to Bayes' rule:

$$Pr(\theta|D) = \frac{Pr(\theta)Pr(D|\theta)}{Pr(D)} \quad (1)$$

$\mathrm{Pr}(\theta)$ denotes he prior probability of observing a specific set of parameters, while $\mathrm{Pr}(\mathrm{D}|\theta)$ denotes the likelihood of observing the data D given the model parameters θ. The probability of observing the data $\mathrm{Pr}(\mathrm{D})$ without the context of a model (or models) is most often unknown. As such samples from the posterior distribution are known in probability often only with relation to other samples.

The probability of observing the data $\mathrm{Pr}(\mathrm{D}|\mathrm{M})$ in a context of a specific model M, used to fit the data, can be calculated by summing up the probability of observing the specific model parameters (*prior*) multiplied by the probability of observing the data conditioned on the model parameters (*likelihood*), across all the parameter values θ:

$$\mathrm{Pr}(\mathrm{D}|\mathrm{M}) = \sum_{\theta} \mathrm{Pr}(\theta)\mathrm{Pr}(\mathrm{D}|\theta) \quad (2)$$

or in a more general continuous notation:

$$\mathrm{Pr}(\mathrm{D}|\mathrm{M}) = \int_{\theta} \mathrm{f}(\theta)\mathrm{f}(\mathrm{D}|\theta)\mathrm{d}\theta \quad (3)$$

Where $\mathrm{Pr}(\mathrm{D}|\mathrm{M})$ is referred to as the *marginal likelihood*, $f(\theta)$ is the *prior distribution* and $f(\mathrm{D}|\theta)$ is the *likelihood function*.

2.3 **Non-Seasonal Migration Model**

We assume that discrete geographic location diffuses along branches of the tree following a continuous time Markov chain (CTMC) process. In this case, non-seasonal migration processes are characterized by a single rate matrix Q:

$$Q = \begin{pmatrix} -\sum_{i\neq 1}^{n} r_{1i} & r_{12} & \cdots & r_{1n} \\ r_{21} & -\sum_{i\neq 2}^{n} r_{2i} & \cdots & r_{2n} \\ \vdots & \vdots & \ddots & \vdots \\ r_{n1} & r_{n2} & \cdots & -\sum_{i\neq n}^{n} r_{ni} \end{pmatrix} \quad (4)$$

where r_{ij} represents the migration rate between location i to location j.

2.4 **Non-Seasonal Migration Model Parameterization and Priors**

Rates are assumed to have an exponential prior $r_{ij} \sim \exp(\lambda)$ with a rate hyperprior parameter λ which is shared across all the rates and is itself exponentially distributed with unit mean $\lambda \sim \exp(1)$.

Note: the rate hyper prior was added at a later stage and is not included in non-seasonal analysis in the main body of the text .

2.5 **Matrix Exponentiation**

Matrix exponentiation is used to convert migration rates, to probability distributions, which concern the state of nodes along the tree. We first focus on processes along individual branches of the phylogenetic tree.
Given a branch connecting parent node x to child node y, $x \to y$, of length t. We assign node x a vector $\bar{p}_x$ which defines its probability of being at state i:

$$Pr(x_{state} = i) = \begin{cases} p_{x1} & i = 1 \\ \vdots & \vdots \\ p_{xn} & i = n \end{cases} \quad (5)$$

We assume, in the simplest case, that states along branches behave as homogeneous Poisson processes with a rate matrix Q, as such the state distribution of node y can be written as:

$$\bar{p}_y = \bar{p}_x \cdot \mathrm{P} \quad (6)$$

Where P is the transition probability matrix and can be calculated as follows:

$$P = \exp(Qt) \quad (7)$$

The matrix exponent can be defined by the Taylor expansion of the exponent function:

$$P = I + \frac{(Qt)}{1!} + \frac{(Qt)^2}{2!} + \frac{(Qt)^3}{3!} + \cdots \quad (8)$$

Multiple alternative algorithms are implemented for matrix exponentiation. Several algorithms were either

imported (JBLAS) or directly implemented in the code including the Taylor Series, the Padé approximate [11], and Eigen-Decomposition. For matrices of rank 3 or less, and for specific cases of matrices of rank 4 (HKY [12], JC69 [13]), analytic solutions exist and were implemented. All matrix exponentiation algorithms were cross-validated within the package.

2.6 Two Seasonal Migration Model

A seasonal migration model which is a variation of [14], was established by using two different migration rate matrices Q_A and Q_B for two parts (seasons) of the year:

$$Q_A = \begin{pmatrix} -\sum_{i\neq 1}^{n} r_{A1i} & r_{A12} & \cdots & r_{A1n} \\ r_{A21} & -\sum_{i\neq 2}^{n} r_{A2i} & \cdots & r_{A2n} \\ \vdots & \vdots & \ddots & \vdots \\ r_{An1} & r_{An2} & \cdots & -\sum_{i\neq n}^{n} r_{ni} \end{pmatrix}, \qquad Q_B = \cdots \tag{9}$$

The exact partitioning of the year is defined by the start ϕ and end ε of season A (without loss of generality). Where: $\phi \in [0,1)$ and $\varepsilon \in [\phi, 1)$.

For example, for $\phi = 0$ and $\varepsilon = 0.25$ the rate matrix Q_A applies to all branch parts within January-March, while rate matrix Q_B applies to all branch parts within April-December. To estimate the transition probabilities between two states at different times, the respective transition probability matrices P_A, P_B are calculated for the individual year parts through matrix exponentiation. For instance, for the same partitioning of the year, given a branch $x \to y$ spanning from year $t_x = 2$ to year $t_y = 2.75$, the state distribution of node y can be calculated as:

$$\bar{p_b} = \bar{p_a} \cdot \mathrm{P} \tag{10}$$

Where P is the transition probability matrix:

$$P = \exp(Q_A \cdot \Delta t_A) \cdot \exp(Q_B \cdot \Delta t_B) \tag{11}$$

$\Delta t_A = 2.25 - 2 = 0.25$ is the fraction of the branch within season A, and $\Delta t_B = 2.75 - 2.25 = 0.5$ is the fraction of the branch within season B.

2.7 Two Seasonal Migration Model Parameterization and Priors

2.7.1 Migration Seasonality Based on a Specific Source and Destination

Migration rates for the two partitions of the year were parameterized as follows:

$$r_{Aij} = r_{ij} \cdot (1 + \sigma_{ij}), \qquad r_{Bij} = r_{ij} \cdot (1 - \sigma_{ij}) \tag{12}$$

Where r_{ij} is referred to as the mean migration rate, and σ_{ij} are referred to as the *seasonal scaling* parameters. As is the case in the non-seasonal model, mean rates are assumed to have an exponential prior $r_{ij} \sim \exp(\lambda)$ with a rate hyper prior parameter λ which is shared across all the rates and is itself exponentially

distributed with unit mean $\lambda \sim \exp(1)$. The *seasonal scaling* parameters $(\sigma_{12}..\sigma_{n-1,n})$ are assumed to have a uniform prior $\sigma_{ij} \sim U(-1,1)$. The scaling parameter was used instead of two separate rates, to separate the inference of mean migration rates, from the inference of the seasonality of migration.

2.7.2 *Migration Seasonality Based on Source*

For source based migration seasonality rates (Equation 9) are parameterized in the following way:

$$r_{Aij} = r_{ij} \cdot (1 + \sigma_{Si}), \qquad r_{Bij} = r_{ij} \cdot (1 - \sigma_{Si}) \tag{13}$$

where σ_{Si} are the source based *seasonal scaling* parameters. Mean rates are assumed to have an exponential prior $r_{ij} \sim \exp(\lambda)$ with a rate hyper prior parameter λ which is shared across all the rates and is itself exponentially distributed with unit mean $\lambda \sim \exp(1)$. The *seasonal scaling* parameters $(\sigma_{S1}..\sigma_{Sn})$ are assumed to have a uniform prior $\sigma_{Si} \sim U(-1,1)$

2.7.3 *Migration Seasonality Based on Destination*

For destination based migration seasonality rates (Equation 9) are parameterized in the following way:

$$r_{Aij} = r_{ij} \cdot \left(1 + \sigma_{Dj}\right), \qquad r_{Bij} = r_{ij} \cdot \left(1 - \sigma_{Dj}\right) \tag{14}$$

where σ_{Dj} are the destination based seasonal scaling parameters. Mean rates are assumed to have an exponential prior $r_{ij} \sim \exp(\lambda)$ with a rate hyper prior parameter λ which is shared across all the rates and is itself exponentially distributed with unit mean $\lambda \sim \exp(1)$. The seasonal scaling parameters $(\sigma_{D1}..\sigma_{Dn})$ are assumed to have a uniform prior $\sigma_{Dj} \sim U(-1,1)$.

2.7.4 *Migration Seasonality Based on Source and on Destination*

For destination based migration seasonality rates (Equation 9) are parameterized in the following way:

$$r_{Aij} = r_{ij} \cdot (1 + \sigma_{Si}) \cdot \left(1 + \sigma_{Dj}\right), \qquad r_{Bij} = r_{ij} \cdot (1 - \sigma_{Si}) \cdot \left(1 - \sigma_{Dj}\right) \tag{15}$$

where σ_{Si} and σ_{Dj} are the source and destination based seasonal scaling parameters respectively. Mean rates are assumed to have an exponential prior $r_{ij} \sim \exp(\lambda)$ with a rate hyper prior parameter λ which is shared across all the rates and is itself exponentially distributed with unit mean $\lambda \sim \exp(1)$. The *seasonal scaling* parameters $(\sigma_{S1}..\sigma_{Sn},\ \sigma_{D1}..\sigma_{Dn})$ are assumed to have a uniform prior or $\sigma_{Sj} \sim U(-1,1)$ and $\sigma_{Di} \sim U(-1,1)$

2.8 **Tree Likelihood Calculation**

Given a tree, a specific and parameterized trait evolutionary (substitution) model, and the state of traits on the tips of the tree, a tree likelihood can be calculated [6].

In general, this likelihood can be calculated by integrating (enumerating and summing up) the likelihood of all possible internal node states. This is done efficiently by calculating and storing the likelihood of sub-trees, recursively progressing from the tips towards the trunk of the tree [6].

The transition probability matrix is defined according to Equation 7 for a non-seasonal model and according to Equation 11 for a two seasonal model. The transition probability matrix is used to calculate the likelihood of node states along individual branches of the tree.

The prior assumption $\bar{\pi}$ about the state of the root of the tree usually follows either an equal probability of being at each state, an empirical estimate of being at a given state, or the stationary distribution of the substitution model:

$$\bar{\pi} = \bar{p}_0 \cdot \lim_{t\to\infty} \exp\,(Qt) \tag{16}$$

where $\bar{p}_0$ is the initial state of the system and assumed to be an equal probability of being in each state The value of $\bar{p}_0$ is only relevant if isolated populations exist, and stationary conditions depend on their populace.

Since there is no such stationary distribution for a seasonal model, we used the stationary distribution of the corresponding seasonal migration matrix at the root node time, this assumes some convergence to the stationary distribution within each season. Alternative estimates can be derived. The inference is not sensitive to the specific root prior assumptions in this case.

2.9 Stochastic Mapping

Stochastic mapping is an additional step following the calculation of tree likelihood and ancestral state reconstruction at the nodes of a tree. This mapping allows us to generate a stochastic realization of the state of branches along the tree, in addition to the state of internal nodes, and in so doing, provides samples of migration and mutation events, and their timing along the tree that lead to the observed tip states. Stochastic mapping of both sequence (nucleotide) and character (*e.g.* geographic) annotations is available in *SeasMig*, together with the option of incorporating seasonal migration models. Stochastic mapping is implemented directly in our code based on [8]. Improved performance could be achieved using [10].

A given type of event, migration or mutation, is assumed to behave as a Poisson process along a branch with a rate matrix Q:

$$\mathrm{Q}=\begin{pmatrix} -\sum_{i\neq 1}^{n} r_{1i} & r_{12} & \cdots & r_{1n} \\ r_{21} & -\sum_{i\neq 2}^{n} r_{2i} & \cdots & r_{2n} \\ \vdots & \vdots & \ddots & \vdots \\ r_{n1} & r_{n2} & \cdots & -\sum_{i\neq n}^{n} r_{ni} \end{pmatrix} \tag{17}$$

As such, the timing of the next event given the present state follows an exponential distribution with the rate parameter $\lambda_\mathrm{x} = \sum_{i\neq x}^{n} r_{xi}$, where *x* is the present character state.
Once the timing of the next event is determined, it is chosen based on its relative probability compared to other transition (emigration) events:

$$Pr(y = i) = \begin{cases} \frac{r_{x1}}{\lambda_\mathrm{x}} & i = 1 \\ \vdots & \vdots \\ \frac{r_{xn}}{\lambda_\mathrm{n}} & i = n \end{cases} \tag{18}$$

Given a branch connecting parent node x to child node y, defined to span from time t_x to time t_y, and with ancestrally reconstructed states s_x and s_y respectively. Stochastic events are generated starting from s_x, repeatedly until the state of node y is correctly reconstructed. That is, until an event prior to the timing of node y results in the state s_y, and an additional event time generated is timed to be beyond t_y.

Branch reconstructions that span across seasons were performed by stochastically reconstructing the state between the seasons' boundaries using the initial migration matrix, and by continuing the stochastic mapping forward using the second seasonal matrix and so forth. This is process is reinitialized from node x, until the state of the node y is correctly mapped. The validity of these processes relies on the memory less nature of the Poisson process.

2.10 Markov-Chain Monte Carlo (MCMC)

Markov-chain Monte Carlo, or MCMC, is an algorithm that allows sampling from analytically intractable distributions. Such distributions include the distribution of tree likelihoods given a mutational or a migration model.

The general idea of an MCMC method is to set up a sequence of dependent samples $\theta_1, \theta_1, \ldots$ that is guaranteed to converge to a target distribution, in this case the posterior distribution of our model. In the Metropolis-Hastings algorithm, a change is proposed to the current state, drawn from a *proposal distribution* over possible changes $q(\theta \to \theta^*)$. This change is either rejected, in which case the current sample is repeated, or the proposed change is accepted as the new sample. The Metropolis-Hastings acceptance probability [15, 16]:

$$r(\theta \to \theta^*) = min\left\{1, \frac{f(\theta^*)q(\theta \to \theta^*)}{f(\theta)q(\theta \to \theta^*)}\right\} \quad (19)$$

guarantees that the sequence of samples will converge to the *posterior distribution*.

2.11 Metropolis-coupled MCMC (MC3)

We use (http://github.com/edbaskerville/mc3kit) for MCMC functionality [1, 2]. Additional functions for sampling and evaluating tree likelihoods were implemented.

Although the Metropolis-Hastings algorithm is guaranteed to converge to the target distribution at some point, local maxima in the likelihood surface can cause a chain to become stuck for long periods of time. One approach to avoiding this problem, known as "Metropolis coupling", involves running multiple chains in parallel. One chain, the "cold chain", explores the target distribution, while the other chains, "hot chains", explore low-likelihood configurations more freely. Periodically, swaps are proposed between chains, allowing good configurations discovered on hot chains to propagate toward to the cold chain.

Rather than exploring the target distribution $f(\theta|D) \propto f(\theta)f(D|f(\theta|D)$, heated chains explore

$$f_\tau(\theta|D) \propto f(\theta)[f_\tau(D|\theta)]^\tau, \tau \in [0,1] \quad (20)$$

Where τ is a heating parameter. We use uneven spaced values of τ [17], with the hottest chain exploring the prior ($\tau = 0$) and the coldest chain exploring the posterior ($\tau = 1$).

Swap moves are standard Metropolis-Hastings proposals, but rather than considering a change to a single chain, they consider a change to the joint distribution of two chains. The acceptance proability is thus the ratio of the joint distribution after and before the move:

$$r\left(\left(\theta_i, \theta_j\right) \rightarrow \left(\theta_j, \theta_i\right)\right) = \frac{f\left(\theta_j\right)\left[f\left(D\middle|\theta_j\right)\right]^{\tau_i} f(\theta_i)[f(D|\theta_i)]^{\tau_j}}{f\left(\theta_j\right)[f(D|\theta_i)]^{\tau_i} f\left(\theta_j\right)\left[f\left(D\middle|\theta_j\right)\right]^{\tau_j}} = \left[\frac{f(D|\theta_i)}{f(D|\theta_j)}\right]^{\tau_j - \tau_i} \tag{21}$$

Where θ_i, θ_j are the configurations that begin in chains i and j, and τ_i, τ_j are the heat parameters of the two chains.

The use of multiple heated chains has the side effect of drastically improving estimates of marginal likelihoods for model selection, as described in the next section.

2.12 Marginal Likelihood Estimation

Enumeration across all possible model parameters is computationally costly and grows exponentially with the number of model parameters. We would like to use MCMC to estimate the marginal likelihood for the sake of comparison among different models. Marginal likelihood estimates derived from a single chain, such as the harmonic mean estimator of Raftery [18], converge very slowly, because MCMC fails to sample sufficiently from low-likelihood areas. However, it is possible to use the information gathered about low-likelihood areas in heated chains using a technique called thermodynamic integration [19, 20], or path sampling [21].

Assuming a continuum of heated chains, the thermodynamic estimator of the log-marginal likelihood is:

$$log\hat{\mathcal{L}}(M) = \int_0^1 \frac{1}{m} \sum_{i=1}^{m} \pi\left(\theta_{i,\tau}\right) log\ \mathcal{L}\left(\theta_{i,\tau}\right) d\tau \tag{22}$$

where m is the number of samples in the MCMC output, and $\theta_{i,\tau}$ is a single sample from the output in a chain with heat parameter τ [20]. With a finite number of chains, we use the trapezoid rule to numerically integrate this integral (Figure 1), using uneven spacing of heats to improve the estimate [17].

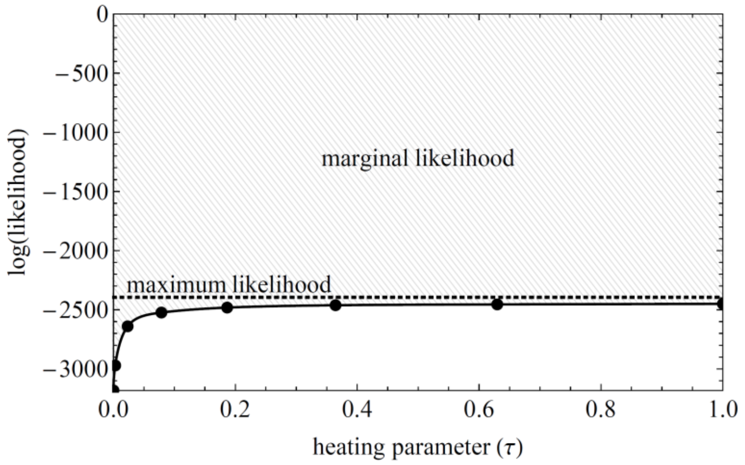

Figure 1. Thermodynamic integration of the marginal likelihood. The mean likelihoods of each chain (black dots) are interpolated and used to estimate the marginal likelihood (gray area) [17]. The maximum likelihood (dotted line) is asymptotically approaches the mean likelihood as $\tau \rightarrow \infty$.

2.13 Model Selection via Marginal Likelihood

The Bayesian framework provides a natural way to make probabilistic inferences based on a particular model. However, we also want to be able to choose between different models by quantifying their relative goodness of fit. One approach to Bayesian model selection can be framed directly in terms of Bayes' rule, mirroring the process for estimating the posterior distribution over parameters for a single model.

Consider two models, M_1 and M_2, to which we assign prior weight $Pr(M_1)$ and $Pr(M_2)$. After the data has been observed, we can calculate the posterior probability of the models using Bayes' rule:

$$\Pr(M_1|D) = \frac{\Pr(M_1)\Pr(D|M_1)}{\Pr(D)} \qquad (23)$$
$$\Pr(M_2|D) = \frac{\Pr(M_2)\Pr(D|M_2)}{\Pr(D)}$$

Where the denominator is equal to the probability of observing the data unconditional of the particular model at play, $\Pr(D) = \Pr(M_1)\Pr(D|M_1) + \Pr(M_2)\,P(D|M_2)$. The probabilities $\Pr(D|M_1) = \int_{\theta_1} f(\theta_1) f(D|\theta_1) d\theta_1$ and $\Pr(D|M_2) = \int_{\theta_2} f(\theta_2) f(D|\theta_2) d\theta_2$ are the marginal likelihoods of the two models, corresponding to Equation 3. If we give the two models equal prior weight, then the relative posterior weight of the two models is simply given by the marginal likelihoods. This reasoning extends naturally to any number of models.

The ratio of the marginal likelihoods is often called the Bayes factor [18, 22, 23], and is equal to the posterior odds ratio of the two models, assuming equal prior weight:

$$B_{12} = \frac{\Pr(D|M_1)}{\Pr(D|M_2)} \qquad (24)$$

The Bayes factor provides a convenient way to compare models: if B_{12}=10, then we consider support for model M_1 to be ten times stronger than model M_2. In AIC-based selection, the Bayes factor is analogous to a ratio of Akaike weights [24].

The marginal likelihood of a a model is the likelihood averaged over the prior distribution. That is, it is the likelihood one would expect by randomly sampling parameters from the prior distribution:

$$f(D|M) = \int_{\theta} f(\theta)f(D|\theta)d\theta \tag{25}$$

This value serves as a useful measure of model fit because it directly incorporates the dependence of the likelihood on uncertainty in parameter values, implicitly penalizing extra degrees of freedom [25]. If an additional parameter improves the maximum likelihood but decreases the average likelihood, the model suffers from over fitting relative to the simpler model.

2.14 **Convergence**

Methods for estimating model convergence were not directly implemented within our package. Such tools include [26] which can be used to estimate the number of effective number of samples from an MCMC chain. This is necessary since MCMC chains include auto correlated samples.

2.15 **Variable Selection**

To assess whether the inclusion of migration between different communities is informative, and to establish if rates are seasonal, Bayesian variable selection [27] was implemented.

Our implementation is based on [28] but differs in that it is implemented within an MC3 framework. Indicator variables which can take a value of either 0 or 1 prefix parameters of interest. Bayes factors for the inclusion of a specific parameter are calculated as:

$$BF = \frac{\Pr(D|M, I = 1)}{\Pr(D|M, I = 0)} \tag{26}$$

and represent the ratio of the marginal likelihoods of the two models, with and without the variable of interest parameterized. Symmetric non-informative priors were used for the indicators. Bayes factors are estimated as the ratio of the number of posterior samples of the cold chain in which the indicator was 1 compared to 0. The use of an MC3 framework reduces the probability of variables getting stuck in a specific configuration (on or off) as heated chains continue to sample from the prior and flattened likelihood distributions. In theory, it may be possible to use thermodynamic integration to obtain better estimates of Bayes factors.

2.16 **Non-Seasonal Migration Model Parameterization with Variable Selection**

Rates (Equation 4) are parameterized in the following way $r_{ij} = \tilde{r}_{ij} \cdot I_{ij}$ where $\tilde{r}_{ij} \sim \exp(\lambda)$ have an exponential prior with a rate hyper prior parameter λ which is shared across all the rates and is itself exponentially distributed with unit mean $\lambda \sim \exp(1)$. The indicators I_{ij} are drawn from an equal probability prior distribution.

Note: a rate hyper prior was added at a later stage and is not included in non-seasonal analysis in the main body of the text.

2.17 Two-Seasonal Migration Model Parameterization with Variable Selection

Rates (Equation 9) are parameterized in the following way:

$$r_{Aij} = r_{ij} \cdot I_{Rij} \cdot \left(1 + \sigma_{ij} \cdot I_{\sigma ij}\right), \qquad r_{Bij} = r_{ij} \cdot I_{Rij} \cdot \left(1 - \sigma_{ij} \cdot I_{\sigma ij}\right) \tag{27}$$

where r_{ij} is referred to as the mean migration rate, and σ_{ij} are referred to as the *seasonal scaling* parameters. As is the case in the non-seasonal model, mean rates are assumed to have an exponential prior $r_{ij} \sim \exp(\lambda)$ with a rate hyper prior parameter λ which is shared across all the rates and is itself exponentially distributed with unit mean $\lambda \sim \exp(1)$. The *seasonal scaling* parameters $(\sigma_{12} .. \sigma_{n-1,n})$ are assumed to have a uniform prior $\sigma_{ij} \sim U(-1,1)$. The *seasonal scaling* indicators $I_{\sigma ij}$, and the rate indicators I_{Rij} are drawn from an equal probability prior distribution.

2.18 Combining the likelihood of multiple protein trees

A conservative approach was used to combine the information present in multiple protein trees with respect to the model likelihood. The combined protein tree log-likelihood is averaged across the multiple protein trees, to account for the possible lack of independence in the information contained in the two trees with respect to migration rates and seasonality. This choice does not affect the maximum likelihood model parameter choice but has the effect of widening confidence intervals when the multiple protein trees provide independent data, while providing the correct confidence interval when the proteins are in complete linkage and have the exact same evolutionary history. Tree weights can be specified as configuration parameters.

3 Results

3.1 Inference of non-seasonal and seasonal migration rates

In this analysis we infer seasonal and non-seasonal migration rates from a single tree topology and stochastically generated tip locations based on a known input migration model. A single hemagglutinin tree topology with 2859 tips was used for this analysis. Tip collection dates span from 1981-2009. Non-seasonal and two-seasonal migration models without variable-selection are used in this section.

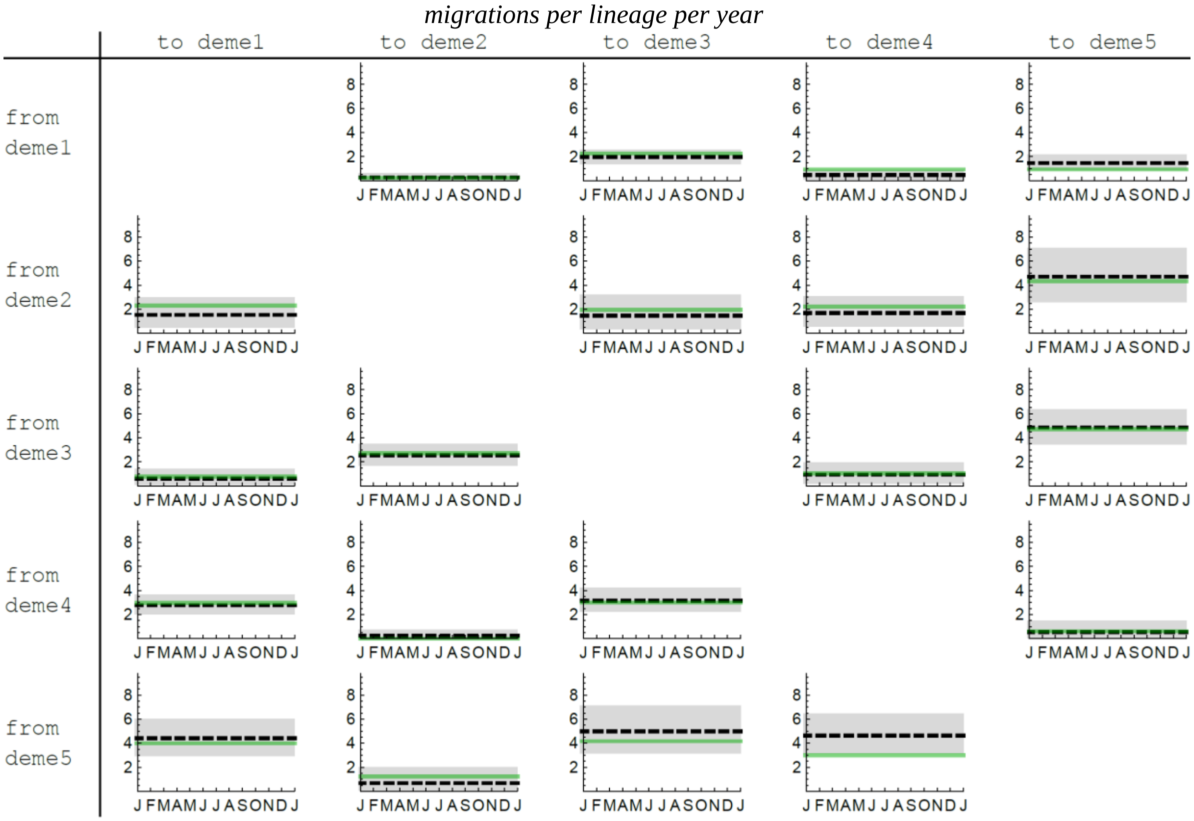

Figure 2 Inferred migration rates given a non-seasonal tip location generating model Inferred median (dashed) and 90% Bayesian credible intervals (gray) (CI) for migration rates between five locations. Tip locations were generated stochastically using an input non-seasonal migration model (green line). A non-seasonal migration rate model was used for inference.

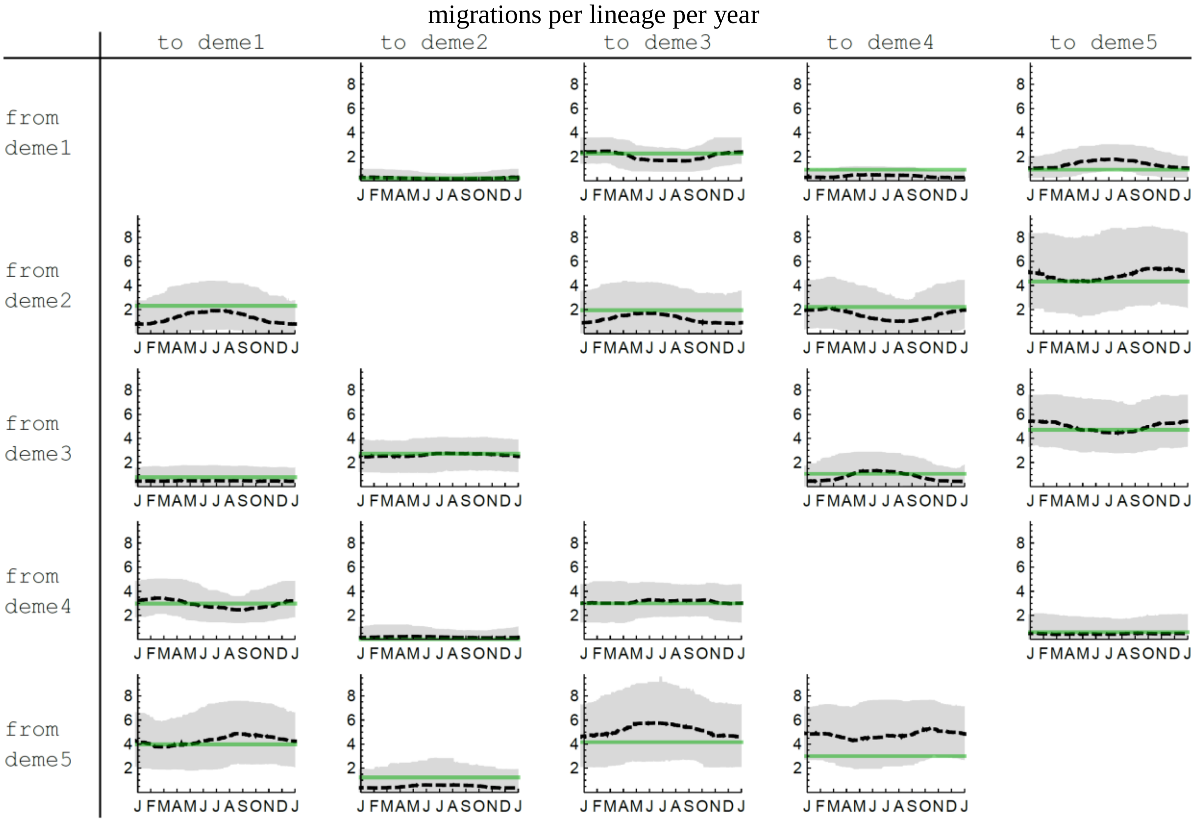

Figure 3 Inferred migration rates given a non-seasonal tip location generating model Inferred median (dashed) and 90% CI (gray) migration rates between five locations. Tip locations were generated stochastically using an input non-seasonal migration model (green line). A two-seasonal migration rate model was used for inference.

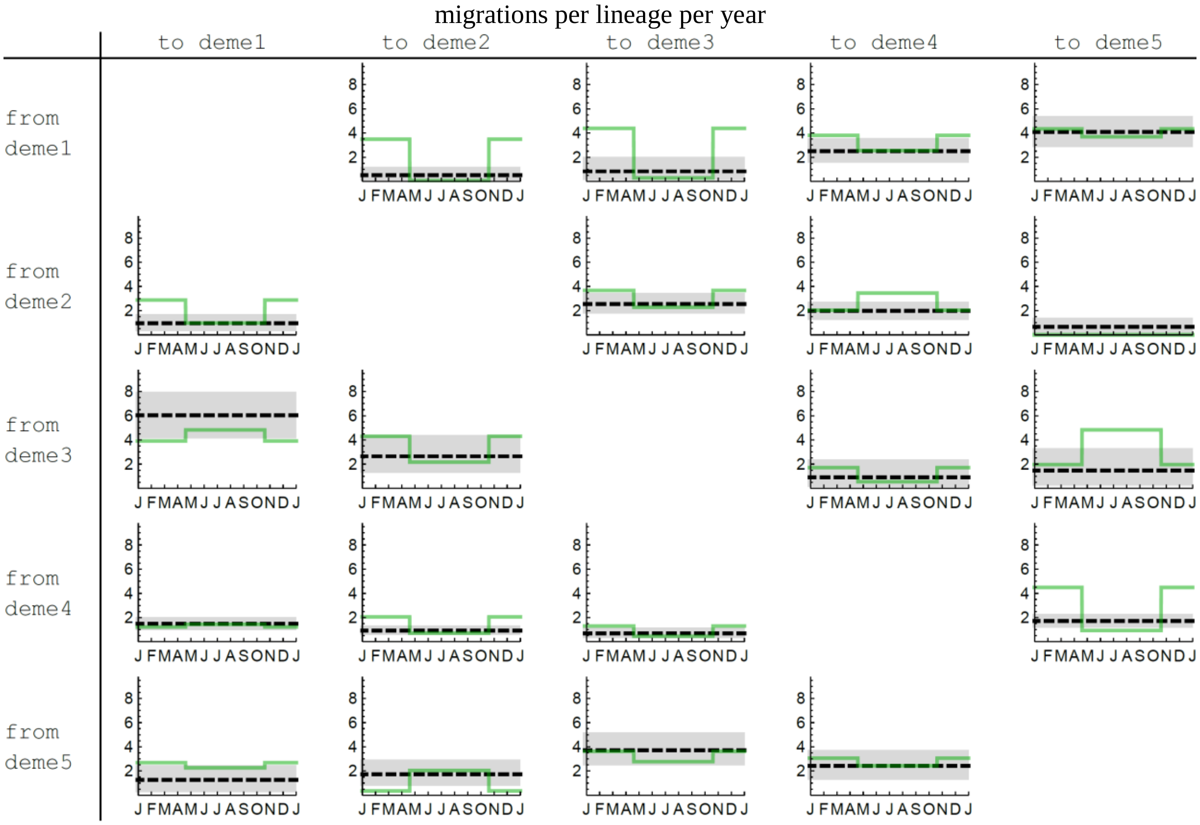

Figure 4 Inferred migration rates given a two-seasonal tip location generating model Inferred median (dashed) and 90% CI (gray) migration rates between five locations. Tip locations were generated stochastically using an input two-seasonal migration model (green line). A non-seasonal migration rate model was used for inference.

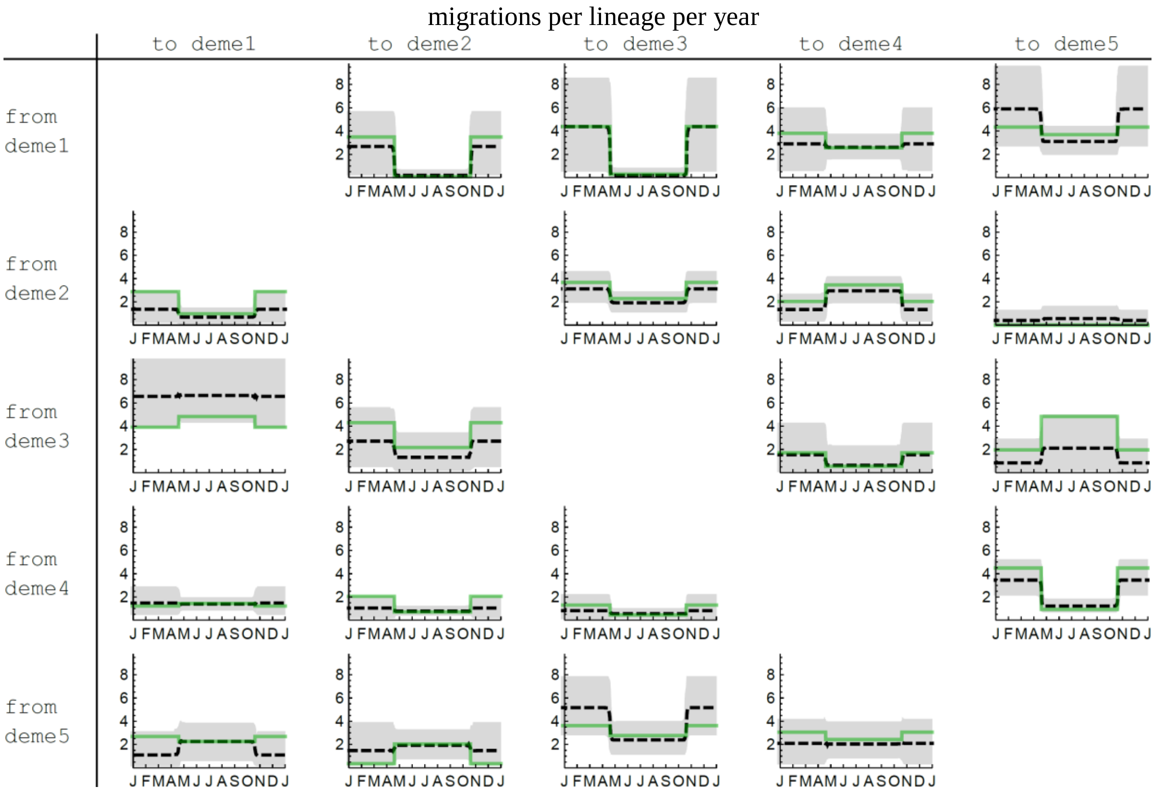

Figure 5 Inferred migration rates given a two-seasonal tip location generating model Inferred median (dashed) and 90% CI (gray) migration rates between five locations. Tip locations were generated stochastically using an input two-seasonal migration model (green line). A two-seasonal migration rate model was used for inference.

When using the input migration model type (seasonal vs. two-seasonal), migration rates are mostly, but not always within the 90% Bayesian credible intervals.

3.2 Marginal likelihood of non-seasonal and seasonal migration models

We compare the marginal likelihood of alternative non-seasonal and two-seasonal migration models. For this tree and the specified input models (Table 1). The correct migration model (seasonal vs. non-seasonal) is supported for 3, 5 and 8 demes based on the marginal likelihood.

Table 1. Marginal likelihood of seasonal and non-seasonal migration rate models

# demes	tip generating model	inference model	marginal likelihood
3	non-seasonal	non-seasonal	-2370.4
3	non-seasonal	two-seasonal	-2374.2
3	two-seasonal	non-seasonal	-2491.3
3	two-seasonal	two-seasonal	-2478.9
5	non-seasonal	non-seasonal	-3899.3
5	non-seasonal	two-seasonal	-3914.7
5	two-seasonal	non-seasonal	-4016.4
5	two-seasonal	two-seasonal	-3979.7
8	non-seasonal	non-seasonal	-5055.2
8	non-seasonal	two-seasonal	-5069.6
8	two-seasonal	non-seasonal	-5494.4
8	two-seasonal	two-seasonal	-5430.4

3.3 Inference of seasonal migration from simulated trees

3.3.1 Five populations, single protein tree

We used an agent based simulation [29] to simulate migration between different populations with random population size, associations and seasonal incidence patterns (Table 2, Table 3, Figure 6). In this model the number of infected contacts between *deme i* and *deme j* was drawn from a Poisson distribution with mean:

$$contacts_{i \to j} = \beta_0 \cdot I_i(t) \cdot \frac{S_j(t)}{N_j} \cdot c_{i \to j} \cdot s_i(t) \tag{28}$$

where β_0 is the contact rate, $I_i(t)$ the number of infected at the source deme, $S_j(t)$ the number of susceptible at the destination deme, N_j the population size of the destination deme, $c_{i \to j}$ is the fraction of contacts between the demes (Table 3) as part of within deme contact, and $s_i(t)$ is the seasonality in the contact rate at the source deme (an alternative could be at the destination deme contact seasonality).
Infection is further determined by the immune history of the host and the cross-immunity with the infecting strain. As such none of these parameters can be directly associated with migration rates on a per lineage basis.

Table 2 Agent based five deme population parameterization

Parameter	Value
contact rate β_0	0.6 [1/day]
recovery rate ν	0.2[1/day]
birth/death rate μ	1/30 [1/year]
epitopes	4
variants per epitope	5x4x3x2
epitope mutation rate ξ	0.000008 [1/day]
cross-immunity σ	0.87

A single tree with ~3000 tips tracking the genealogy of the simulated virus was sampled proportional to the prevalence.

Table 3. Random associations $(c_{i \rightarrow j})$ between five simulated populations as a fraction of within deme contact

from/to	deme1	deme2	deme3	deme4	deme5
**deme1**		0.007	0.039	0.039	0.041
**deme2**	0.045		0.005	0.005	0.031
**deme3**	0.014	0.032			0.013
**deme4**	0.007	0.029	0.033	0.033	0.014
**deme5**	0.042	0.040	0.011	0.011	

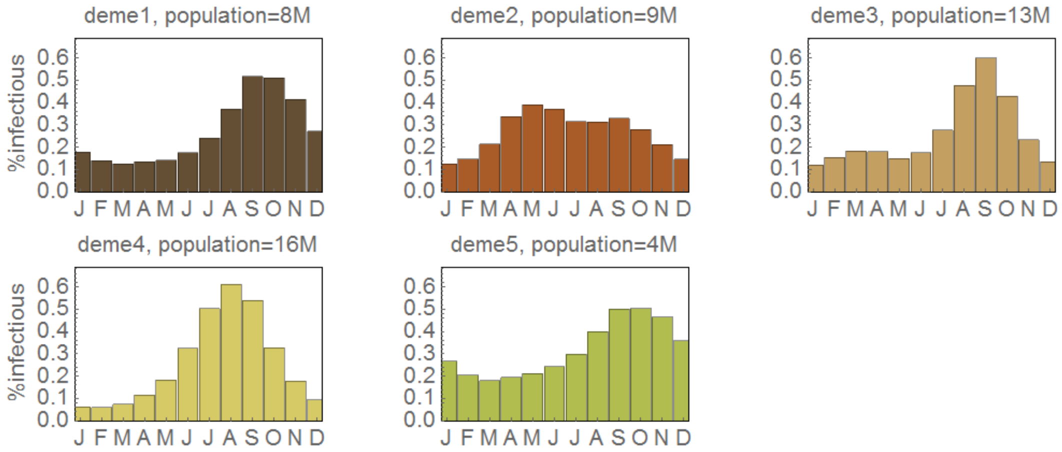

Figure 6 Prevalence seasonality in simulated population Simulation of 5 populations with seasonal incidence patterns and random associations.

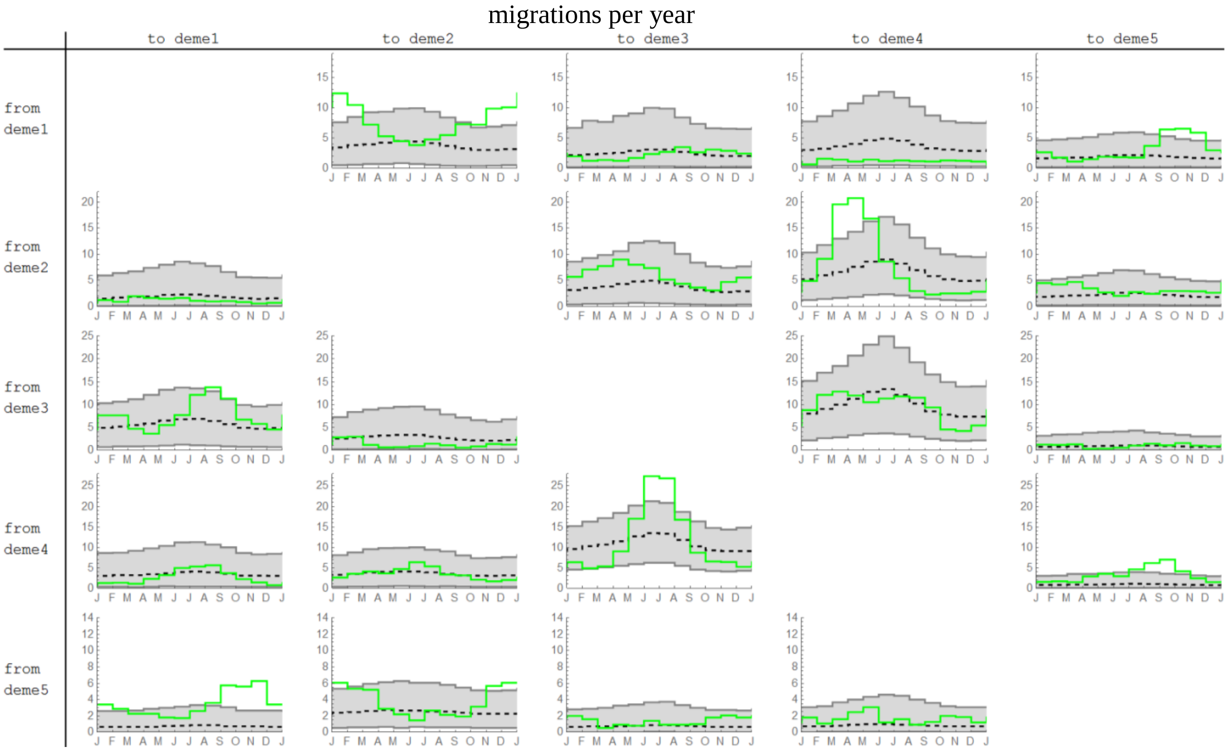

Figure 7 Inferred migration events seasonality using a constant migration model Inferred median (dashed) and 90% CI (gray) stochastically mapped migrations between five locations. Samples of the migration events on the simulation tree (green line). A model with constant migration rates is used (marginal likelihood = -4593.7).

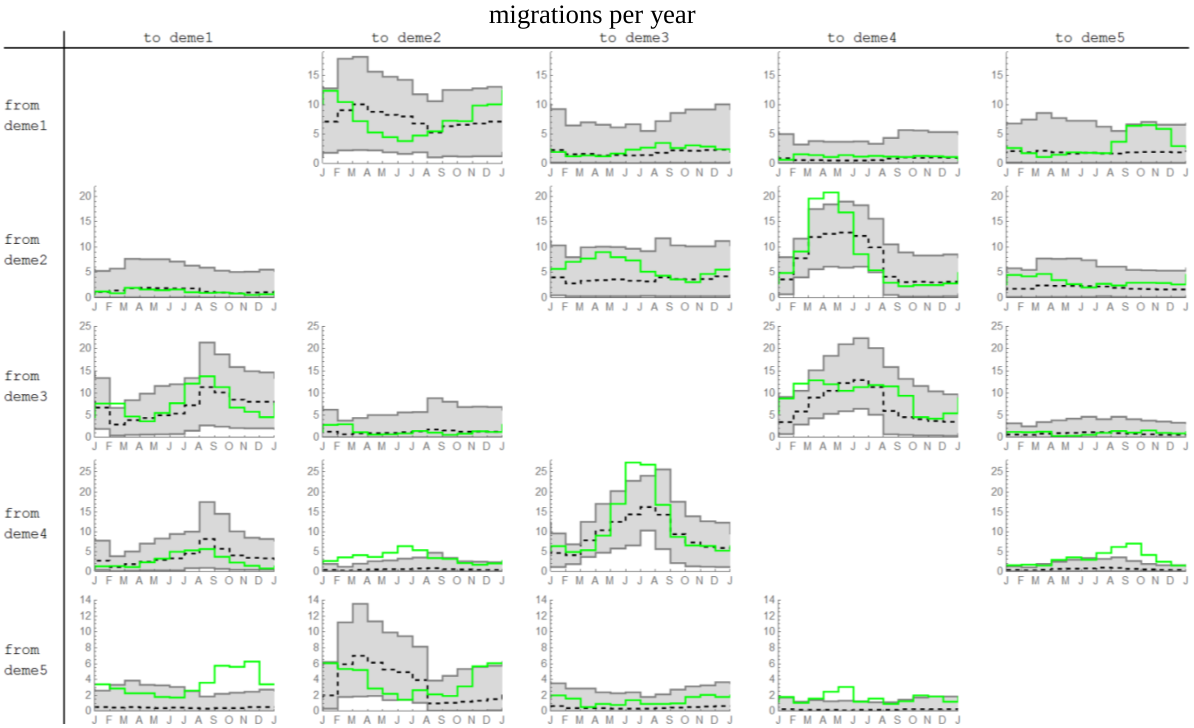

Figure 8 Inferred migration events seasonality using a two-seasonal migration model Inferred median (dashed) and 90% CI (gray) stochastically mapped migrations between five locations. Samples of the migration events on the simulation tree (green line). A two seasonal migration model is used (marginal likelihood = -4510.2)

3.3.2 Three populations, two protein trees

We simulated [29] migration between three different seasonal populations with a specified population size. Contact seasonality (Figure 9, observed seasonality) and a associations (Table 4) were randomly parameterized. A limited number of tip samples were used to sample the transmission tree as specified by Table 5 intended to approximate the sampling profile of the main text, and Table 6 representing uniform sampling over time. Each simulation was repeated twice to attain to alternative evolutionary histories (proteins) driven by the same migration process. Future simulations will include the direct simulation of segmented genome viral evolution.

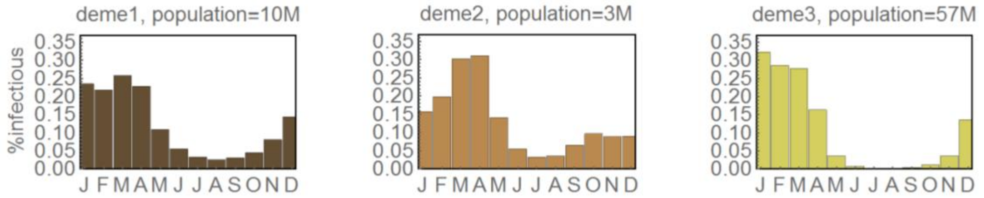

Figure 9. Prevalence seasonality in simulated population Simulation of three populations with seasonal incidence patterns and random associations.

Table 4. Random associations ($c_{i \to j}$) between three simulated populations as a fraction of within deme contact

from/to	deme1	deme2	deme3
**deme1**		0.007	0.039
**deme2**	0.045		0.005
**deme3**	0.014	0.032	

3.3.2.1 Uniform sampling over time

In this simulation, we sampled approximately the same number of tips over time irrespective of population size and of seasonal incidence patterns.

Table 6. Number of tips samples from two simulated protein trees with alternative evolutionary histories and the same underlying migration processes. Tips were sampled stochastically, with approximately the same number of tips sampled over time and in each population.

	protein A	protein B	Total
**deme1**	287	278	565
**deme2**	252	273	525
**deme3**	290	297	587
**Total**	829	848	1677

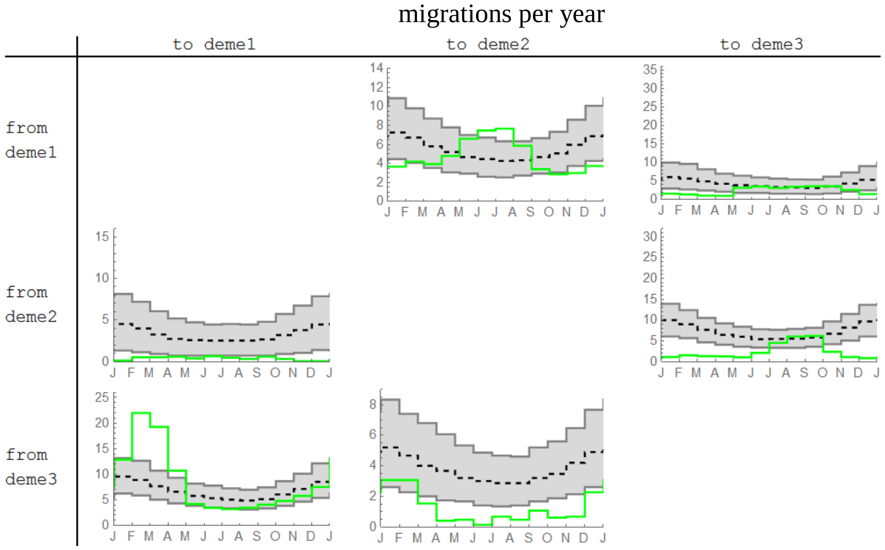

Figure 10 Inferred migration events seasonality using a non-seasonal migration rate model and uniform tip sampling Inferred median (dashed) and 90% CI (gray) stochastically mapped migrations between five locations. Samples of the migration events on the simulation tree (green line). A two seasonal migration model is used (marginal likelihood = -902.3)

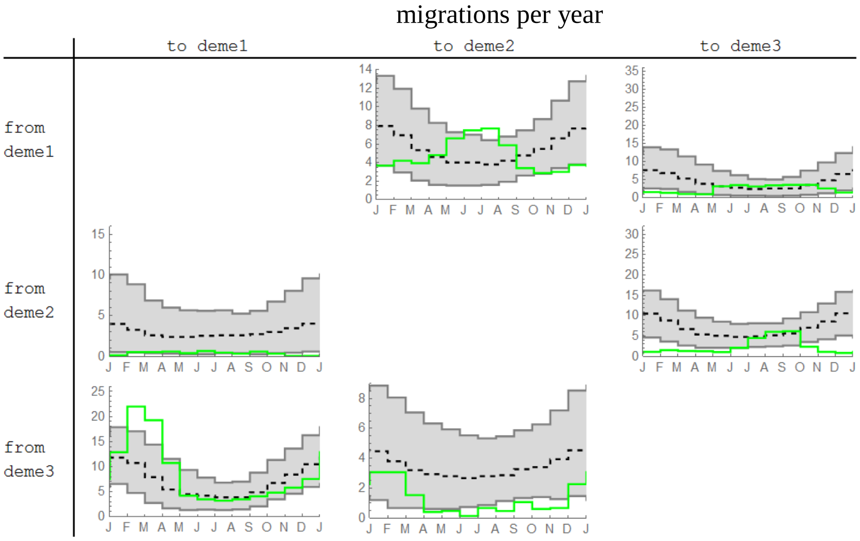

Figure 11 Inferred migration events seasonality using a two-seasonal migration rate model and uniform tip sampling Inferred median (dashed) and 90% CI (gray) stochastically mapped migrations between five locations. Samples of the migration events on the simulation tree (green line). A two seasonal migration model is used (marginal likelihood = -906.9)

3.3.2.2 Proportional sampling

In this simulation tips were sampled proportional to incidence. The number of tips (Table 5) sampled is intended to approximate the available hemagglutinin and neuraminidase sequences which were used in the main text for a similar inference.

Table 5. Number of tips samples from two simulated protein trees with alternative evolutionary histories and the same underlying migration processes. Tips were sampled stochastically, proportional to prevalence, with an approximate number of tips specified in each population.

	**protein A**	**protein B**	**Total**
**deme1**	514	47	561
**deme2**	138	2	140
**deme3**	1369	323	1692
**Total**	2021	372	2393

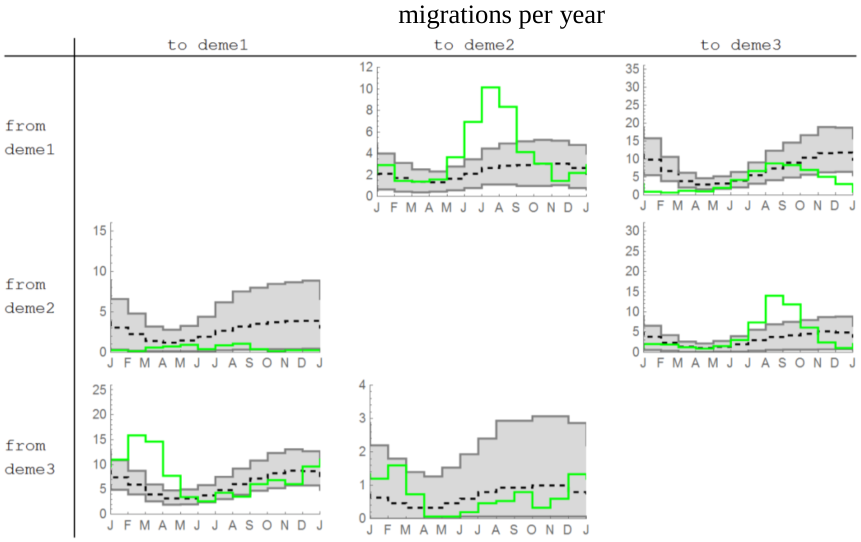

Figure 12 Inferred migration events seasonality using a constant migration rate model Inferred median (dashed) and 90% CI (gray) stochastically mapped migrations between five locations. Samples of the migration events on the simulation tree (green line). A model with constant migration rates is used (marginal likelihood = -899.3).

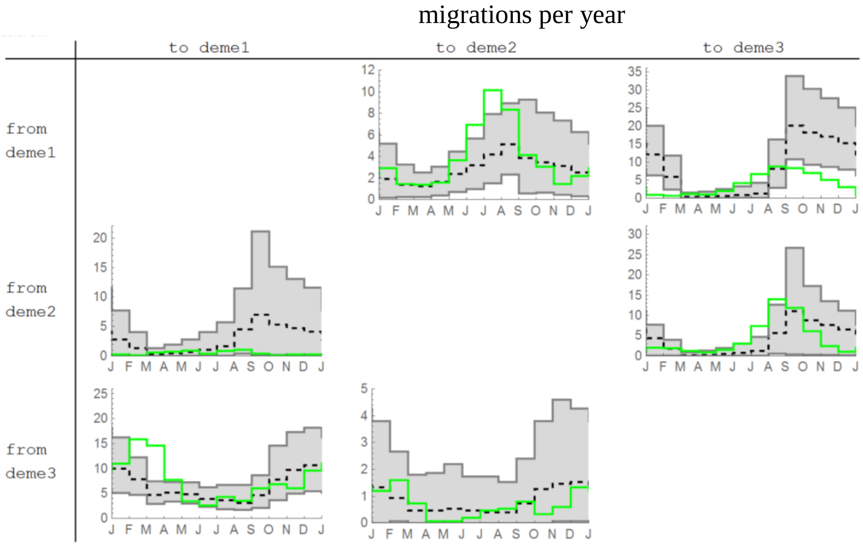

Figure 13 Inferred migration events seasonality using a two-seasonal migration rate model Inferred median (dashed) and 90% CI (gray) stochastically mapped migrations between five locations. Samples of the migration events on the simulation tree (green line). A two seasonal migration model is used (marginal likelihood = -862.7)

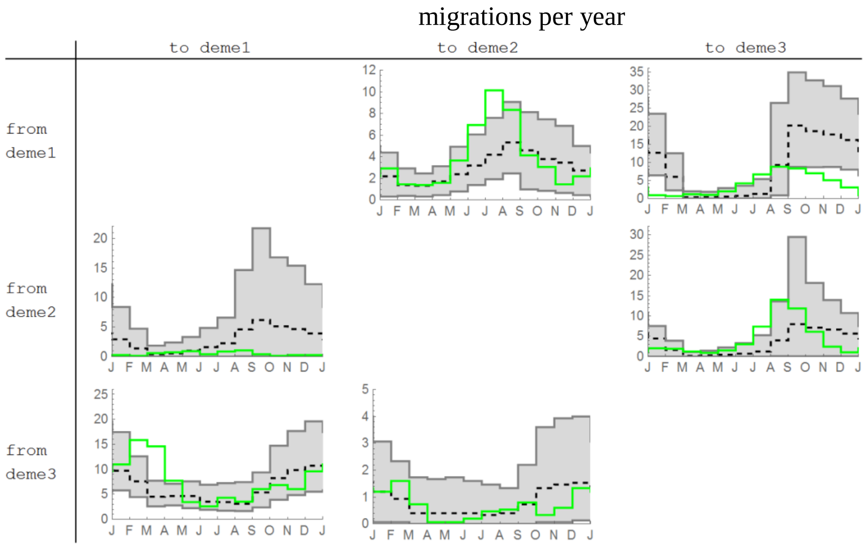

Figure 14 Inferred migration events seasonality using a two-seasonal migration rate model with variable selection for the inclusion of migration between deme pairs and for the inclusion of seasonal migration between deme pairs Inferred median (dashed) and 90% CI (gray) stochastically mapped migrations between five locations. Samples of the migration events on the simulation tree (green line). A two seasonal migration model is used (marginal likelihood = -866.5)

4 References

1. Baskerville, EB, Dobson, AP, Bedford, T, Allesina, S, Anderson, TM, Pascual, M: **Spatial Guilds in the Serengeti Food Web Revealed by a Bayesian Group Model**. *PLoS Comput Biol* 2011, **7**:e1002321.

2. Baskerville EB, Bedford T, Reiner RC, Pascual M: **Nonparametric Bayesian grouping methods for spatial time-series data**. *arXiv preprint arXiv:13065202* 2013.

3. Drummond AJ, Suchard MA, Xie D, Rambaut A: **Bayesian phylogenetics with BEAUti and the BEAST 1.7**. *Molecular Biology and Evolution* 2012.

4. Huelsenbeck JP, Ronquist F: **MrBayes: Bayesian inference of phylogenetic trees**. *Bioinformatics* 2001, **17**:754 – 755.

5. Pagel M, Meade A, Barker D: **Bayesian Estimation of Ancestral Character States on Phylogenies**. *Systematic Biology* 2004, **53**:673–684.

6. Felsenstein J: **Evolutionary trees from DNA sequences: a maximum likelihood approach**. *J Mol Evol* 1981, **17**:368 – 376.

7. Swofford DL: **{PAUP*. Phylogenetic analysis using parsimony (* and other methods). Version 4.}**. 2003.

8. Bollback J: **SIMMAP: Stochastic character mapping of discrete traits on phylogenies**. *BMC Bioinformatics* 2006, **7**:88.

9. Huelsenbeck JP, Nielsen R, Bollback JP: **Stochastic mapping of morphological characters**. *Syst Biol* 2003, **52**:131 – 158.

10. Minin VN, Suchard MA: **Fast, accurate and simulation-free stochastic mapping**. *Philosophical Transactions of the Royal Society B: Biological Sciences* 2008, **363**:3985–3995.

11. Higham NJ: **The scaling and squaring method for the matrix exponential revisited**. *SIAM review* 2009, **51**:747–764.

12. Beerli P, Felsenstein J: **Maximum-likelihood estimation of migration rates and effective population numbers in two populations using a coalescent approach**. *Genetics* 1999, **152**:763.

13. Jukes T, Cantor C: **Evolution of protein molecules**. *Mammalian Protein Metabolism* 1969:21 – 132.

14. Bielejec F, Lemey P, Baele G, Rambaut A, Suchard MA: **Inferring Heterogeneous Evolutionary Processes Through Time: from Sequence Substitution to Phylogeography**. *Systematic Biology* 2014, **63**:493–504.

15. Metropolis N, Rosenbluth A, Rosenbluth M, Teller A, Teller E: **Equations of state calculations by fast computing machines**. *Journal of Chemistry and Physics* 1953, **21**:1087 – 1092.

16. Hastings W: **Monte Carlo sampling methods using Markov chains and their applications**. *Biometrika* 1970, **57**:97 – 109.

17. Friel N, Pettitt AN: **Marginal likelihood estimation via power posteriors**. *Journal of the Royal Statistical Society: Series B (Statistical Methodology)* 2008, **70**:589–607.

18. Kass RE, Raftery AE: **Bayes Factors**. *Journal of the American Statistical Association* 1995, **90**:773–795.

19. Lartillot N, Philippe H: **Computing Bayes Factors Using Thermodynamic Integration**. *Systematic Biology* 2006, **55**:195–207.

20. Beerli P, Palczewski M: **Unified Framework to Evaluate Panmixia and Migration Direction Among Multiple Sampling Locations**. *Genetics* 2010, **185**:313–326.

21. Calderhead B, Girolami M: **Estimating Bayes factors via thermodynamic integration and population MCMC**. *Computational Statistics & Data Analysis* 2009, **53**:4028–4045.

22. Jeffreys H: **Some Tests of Significance, Treated by the Theory of Probability**. *Mathematical Proceedings of the Cambridge Philosophical Society* 1935, **31**:203–222.

23. Jeffreys H: *Theory of Probability*. Oxford University Press, Oxford UK; 1961.

24. Burnham KP, Anderson DR: *Model Selection and Multimodel Inference: A Practical Information-Theoretic Approach*. Springer; 2002.

25. Bolker BM: *Ecological Models and Data in R*. Princeton University Press; 2008.

26. Rambaut A, Drummond AJ: **Tracer [computer program]**. 2003.

27. O’Hara RB, Sillanpää MJ: **A review of Bayesian variable selection methods: what, how and which**. *Bayesian Analysis* 2009, **4**:85–117.

28. Kuo L, Mallick B: **Variable selection for regression models**. *Sankhyā: The Indian Journal of Statistics, Series B* 1998:65–81.

29. Zinder D, Bedford T, Gupta S, Pascual M: **The Roles of Competition and Mutation in Shaping Antigenic and Genetic Diversity in Influenza**. *PLoS Pathog* 2013, **9**:e1003104.
